# Synthetic Biology: Old and New Dilemmas—The Case of Artificial Life

**DOI:** 10.3390/biotech10030016

**Published:** 2021-07-20

**Authors:** Nikolaos Kolisis, Fragiskos Kolisis

**Affiliations:** 1 School of Law, National and Kapodistrian University of Athens, Solonos 57, 10679 Athens, Greece; 2 Biotechnology Laboratory, School of Chemical Engineering, National Technical University of Athens, Zografou Campus, 9, Iroon Polytechniou str, 15780 Athens, Greece; kolisis@chemeng.ntua.gr

**Keywords:** biotechnology, synthetic biology, system biology, bioethics, synthetic life, ethics

## Abstract

This article aims to examine some of the ethical questions emerging from the use of already existing biotechnological tools and the issues which might occur by synthetic biology’s potential future possibilities. In the first part, the essence of synthetic biology and its relation to the contemporary biotechnological research is analyzed. In the second part, the article examines whether the new biotechnological inventions pose new or revive old moral questions about the ethics of science, engineering, and technology in general. After briefly addressing some of the various issues which are raised by experts, philosophers, but also the general public, concerning synthetic biology in general, it focuses on the topic of “artificial life creation” and presents moral reasons which may or may not allow it. The topic is approached by referring to consequentialist, deontological, but also, virtue theory arguments for and against it and the possibility of a partial permission of “artificial life” experiments, asking whether the benefits outweigh the risks and moral implications is explored. Finally, it proposes an argument in favor of the future exploration of biological innovation, underlying the need for a more balanced access to its beneficial results.

## 1. Introduction

Synthetic biology and its aims has been a subject of discussion among scientists, philosophers and the wider public. Its applications influence our lives, and the orientation of its further development is crucial for the progress of humanity in the following years. As expected by such a “scientific revolution”, its birth and growth has led to the emergence of moral and empirical issues. In this article we will try to approach the nature of synthetic biology and its relation to novel biotechnological methods and aims. We will subsequently try to present some of the arguments raised by its applications, especially the emerging controversial subject of synthetic life. After addressing some of the most common arguments against life synthesis, we propose a way to continue research on artificial life under specific moral and empirical terms.

## 2. The Nature of Synthetic Biology

The successful completion of the Human Genome Project triggered an explosive development of contemporary biological research. Based on its findings it was showed that a human has about 25.000 genes, little more than a chimpanzee and far less than a pine tree (about 100.000). It became evident that the function of living organisms could not be addressed satisfactorily by looking at genes and molecules alone, even if all of them were studied [1]. Consequently, at the dawn of the so-called “metagenomics” era of the 21st century, biological research needed to adopt a stochastic approach instead of the, until then, popular deterministic one [2]. The concept of “Systems Biology”, in other words the study of living organisms in terms of their underlying network structure rather than simply their individual molecular components, emerged, conceiving as a “system” anything from a gene regulatory network to a cell, a tissue, or an entire organism [3,4]. The growth of biological research influenced the technological evolution and vice versa, as it happens when big scientific breakthroughs occur. Technological innovations supported biological research by providing sophisticated and precise apparatus as well as “high throughput techniques”. From that moment, it became obvious that computational approaches are required to handle and interpret the data necessary to understand the complex biological systems. Computational Technology was linked with System Biology [5]. This interconnection with the additional integration of engineering principles gave birth to synthetic biology. Synthetic biology can be considered as a research area in which scientists and engineers try to modify existing organisms by redesigning and synthesizing artificial genes, proteins, and metabolic pathways, as well as complete biological systems [6]. This emerging research field is interdisciplinary and consists of scientific tools and principles taken from biology, chemistry, informatics, engineering, mathematics and computational modeling. Synthetic biology’s main aims are first, to improve our understanding of biological systems, of their complexity and of the properties emerging from their interactions, and second, to make possible the use of organisms—cells and their systems—as “factories” for the production, among others, of drugs, biomedical products like vaccines and diagnostics or new tools for biosecurity, and new “smart materials” with specialized properties. The experts of synthetic biology are aiming not only to provide novel biotechnological applications but also to contribute to the advancement of the science of biology in general.

As it happens with many emerging scientific fields, there is not an explicit and universally accepted definition for synthetic biology. Due to its experimental nature, a functional definition could depend on its expected results and applications, or generally on its basic research aims. For instance, some definitions include: “*Synthetic biology aims to design and engineer biologically based parts, novel devices and systems as well as redesigning existing, natural biological systems*” [7], or “*Synthetic biology is the engineering of biology: the deliberate (re)design and construction of novel biological and biologically based parts, devices and systems to perform new functions for useful purposes, that draws on principles elucidated from biology and engineering*” [8]. A definition which seems to represent in a better way the nature of synthetic biology and to clarify that it is not a novel scientific discipline underlines that: “*Synthetic Biology is a further development and new dimension of modern biotechnology that combines science, technology and engineering to facilitate and accelerate the understanding, design, redesign, manufacture and/or modification of genetic materials, living organisms and biological systems*” [9].

One of the fields which attract the interest of many researchers in the development of synthetic biology is the dynamics of the Synthetic Genome. In the Synthetic Genome research projects, scientists can make use of the wealth of information available about genomics as well as the tools that can be used for their manipulation. Amongst them is the oligonucleotide synthesis or genetic modification of the genome towards the creation of new types of genomes, which could lead to new biotechnological applications. Synthetic biologists use two strategic approaches in their studies: the “top-down” and the “bottom-up”. In the top-down strategy they attempt to re-design existing organisms (a bacterium or a virus) or gene sequences in order to remove the genetic parts which are not necessary for the role this organism is intended to play. Specific genetic parts of them can also be replaced or added in order to give the organisms in question new characteristics and functions. The final goal is to create a “minimum genome” or a “minimal cell” (as simple as possible for its survival), which can be used as a “chassis”, where the new genes will be introduced to change or enrich its biological properties and lead to innovative processes [10]. In this “platform” the addition of synthetic genes, or even a whole synthetic genome, are possible, using genetic codes which could consist of synthetic bases, other than the known four of existing life forms, namely A, T, C and G [5,10].The tools of this strategy are computational and experimental comparative genomics, minimal genomics, synthetic genes, metabolic engineering, new metabolic pathways, genetic circuits, etc.

While top-down synthetic biology in general uses properties from living systems to create something new, in bottom-up synthetic biology, which is significantly more challenging, scientists aim to build living systems from raw materials starting from non-living components. In this approach researchers try to create genetically engineered circuits and switches to turn specific functions “on” and “off” in response to designed stimuli, with an ultimate aim to include them in reconstructed vesicles as protocell- approach and cell-free systems [11,12]. A simple gene circuit comprises of a promoter, a ribosome binding site, the protein coding sequence, and a terminator. The reconstitution of the biological systems is based on the idea of their modularity. Each module—which is considered as the smallest functional entity of a biological system—consists of different building blocks with independent functional bio-parts and bio-devices. In current synthetic biology there is a hierarchy based on (a) the bio-parts, which encode biological functions (e.g., synthetically designed DNA), (b) the bio-devices, which are made from a collection of bio-parts and encode human defined functions (e.g., logic gates), and (c) bio-systems, which perform tasks, such as counting and intracellular control functions. This complex network can be re-designed and reconstructed according to the properties one wants the system under investigation to have [6].

To sum-up, the Systems Biology approach uses quantitative methods and, based on the systems engineering principles and on signal theories, attempts to analyze the biological systems under investigation. From the moment a system can be described in mathematical terms, synthetic biology organizes it into bio-parts or bio-devices and estimates their functionality using the classic reductive method. Following this methodology, complex systems and processes can be synthesized by well characterized, registered, and standardized parts and devices. An ideal objective could be the construction of a synthetic cell—an artificial synthetic life form—which can have various applications, such as the synthesis of products of high added value or can be used as an instrument of high technological specialization in specific applications (for example as biosensors used for the diagnosis of various diseases or in order to control the levels of toxic substances in the environment).

## 3. Critique of Synthetic Life Experiments

Public, philosophical and scientific scepticism towards biotechnological advancements involves opinions which oppose the “substitution” of God or nature by humanity, fear of the potential emergence of reductionist views about life (which may lead to undermining its value and affect the way humanity conceives itself and the environment) and finally, question the moral status of the artificially created life forms. As we have argued continuous innovation in the field of biology and the contribution of sciences like physics, mathematics and computer science rendered biologists capable of creating their own models and to intervene to life forms rather than just observing them. Thus, the drawbacks mentioned are not considered novel. Yet, since the first steps towards life creation by experiments such as the one conducted in the Craig Venter Institute [13], criticism and concerns have been revived. In this part, we will try to address the “playing God”, “undermining life’s value”, “creating organisms of unknown status” arguments. We will explore different aspects of terms such as “living organism”, “artificially created life” and “natural” beings and environment, and will approach according to consequentialist, deontological and virtue theory-based principles the issue of “synthesizing life forms”.

### 3.1. The “Natural”-“Artificial” Dipole

The differences between “top-down” and “bottom-up” approaches have already been analyzed. Top-down processes are characterized by the use of already existing cells. Experiments, such as the one conducted at the Craig Venter Institute which resulted to the creation of “Synthia”, are substituting natural DNA with an artificial. For this reason this procedure is not considered to be a complete life synthesis, it has been characterized as a copy of an already existing organism. Bottom-up experiments on the other hand are intended to create viable organisms from simple matter. Their approach seems to be closer to what we might call “life synthesis” [14]. Before we proceed to the examination of whether the creation of “artificial life” is morally acceptable or not and, if so, under what terms, we need to make a brief assessment on what can be perceived as a living entity.

The question “what is life” has been central since humanity’s first steps in rational thinking. Since the Aristotelian conception of life, there has been significant alteration of the exact meaning of the term. In general, a living being can be defined by its capacity to metabolize, to reproduce and die [15]. An organism can also be conceived as an entity in constant flux. A living being must interact with its environment in order to survive—it needs to get the substances which are essential for its self-preservation. An organism, by interacting with its environment and eventually by dying, becomes a part of the process of evolution [16]. Living organisms are morally important as they have interests; through research and observation one can conceive what is good for them and what makes them flourish and act accordingly. For some thinkers who adopt a bio-centrist approach [17], all living entities matter morally and their interests need to be taken into account when planning our actions. Life synthesis may bring novel moral questions when adding moral agents—organisms with interests—which might be taken under consideration. So far, part of the moral argument in favor of the respectful treatment of organisms, other than human, was our common ancestry through evolution. Synthetic biology might change that by creating artificial life. The question might now be whether artificial beings matter morally.

But where does one draw the line between the “natural” and the “artificial”? The concept of nature and its relationship with humanity—humans in nature—has been the subject of discussion for many thinkers. J.S. Mill has famously approached nature either as: (a) anything that happens in the world or (b) anything that happens without human voluntary causation. Some preservationists aim at conserving the parts of the world which have not been altered definitely by human intervention [18]. A more recent approach, made by K. Soper [19] defines nature in three ways: either as a concept needed for the separation of humanity by its environment; a concept useful for us so that we can think of the distinction between human and non-human, or as the way in which natural sciences interpret and explain what occurs in the world (including human actions). In “lay terms”, this means taking as “natural” anything that is not profoundly human made, such as environments other than cities or factories; this may include non-human animals, forests, etc. Following J.S. Mill, we believe that what is considered “natural” cannot be the guide for human behavior, let alone the basis of moral claims. For this reason, in our opinion, arguments criticizing synthetic biology’s “unnaturalness” need to focus more on the way the procedure is conducted (how scientists conceive of their role, how the created organisms are treated, how respectful for life is the regulation etc. [20]). Secondly, as we tried to show, synthetic biology is not a novel discipline, rather it combines already used methods in order to achieve its aims so far. For example, we find that the procedures and tools used by synthetic biology have not been criticized as immoral when applied in genomics or systems biology.

For some thinkers, living beings are characterized by the fact that they have an inherent purpose—a “telos” or aim to flourish or to satisfy their interests. According to them, a synthetic organism will have both transcendental and immanent aims or, in some other thinkers’ terms, proximate and ultimate interests, thus occupying a position between fully artificial and fully natural beings [21]. Fully artificial beings have no goal separate from that of their user/creator and therefore have only transcendental aims, while fully natural beings—as we believe—have immanent aims naturally emerging through evolution. By immanent or proximate interests, we classify all functions which aim at the conservation or the reproduction of the organism. Therefore, their use by humans must be regulated accordingly [15].

Humans must conceive both their intrinsic and their instrumental value. The lines blur if we consider activities such as animal breeding, which follows a natural process but is human directed; many species would have been completely different had humanity not intervened, shaping them for its own aims. Furthermore humans do create, but they are a part of nature. Humanity is a product of evolution; humans are animals which, by using their evolved capabilities, interacted with the environment in different ways than other animals did in order to achieve self-preservation and conservation of the species. Despite humanity’s achievements, it remains part of nature, so whatever humanity produces could, in this way, be considered natural. Humans obey the laws of physics and are part of the evolutionary process just like every other being on Earth. It can also be noted that if any synthetic life form (even organisms that do not exist in nature, such as XNA-organisms) obey to the same laws of physics, biology etc. and are thought of as “living”, they can be considered also a part of nature. Other thinkers underline that for many years vocabulary used to describe machines was also employed in order to explain biological processes and functions. In these terms, the blurring of the line between “machine” and “organism” or “artificial” and “natural” might not be so obvious, especially in an age of intervention to and manipulation of the genome of natural organisms. On the other hand, an abstract approach of genetically modified life forms, synthetic life forms and “living machines” may prove to be a very slippery slope and prepare a way of unequal treatment of the beings “created” [22].

### 3.2. “Playing God” or “Substituting Nature”

In our opinion, in order to address the problem of the moral status and the treatment of synthetic organisms, we must first ask whether their creation is inherently wrong. We find that the commonly presented and frequently adopted “playing God” or “substituting Nature” argument must be examined under the perspective of previous and future human actions. It is claimed that humans must not mess with certain aspects of nature: that reaching so far into the secrets of life constitutes a hubris, an immoral attitude, or our species tendency towards domination and control [21,23,24]. As humans are neither omnipotent nor omniscient, the consequences of their actions in this field may be proven disastrous for the planet [25]. We believe that although it is true that humans present a destructive tendency to expand and consume the planet’s resources and to mistreat non-human animals, it might be claimed that this tendency is linked to a specific way of organizing our society and developing our economy and not part of our biology. In other words, whether these experiments will end up being another addition in the series of human products is a matter of control and regulation.

As mentioned, humans have always manipulated other life forms in order to ameliorate their own state. Of course, the fact that something has always been this way is not enough to justify anything (the examples of slavery, sex inequality and the current harsh treatment of animals prove so). For this reason, it might be morally sound that humans better abstain from deepening their knowledge in this domain. One could also argue that if there indeed exists a special value in natural organisms and life forms in general, this might be based on the fact that they are products of the evolutionary process—we share with them a common ancestor and we have a genetic proximity. The same cannot be said for organisms that are synthesized in a lab. For this reason, these kind of organisms are different and, as they are manmade, they are inferior.

Contrary to these claims, we find that, the inherent value of a being rests not on the way it is brought to life, but on the properties we choose to attribute to it. As the example of the IVF babies shows, we don’t consider IVF babies to be inferior. If indeed there exists a special value in life it must be conserved and shared by all beings we consider “living”—artificial or natural [26,27]. The fact that science needs to advance humanity’s knowledge on life’s mechanisms and characteristics admits exactly that we are not omniscient and will never be. The way we approach the world around us—the inherent curiosity of mankind— helps us understand and admire its complexity. It is our belief that a push forward towards scientific research expresses exactly this kind of admiration.

### 3.3. Is Synthetic Life Leading to Reductionism?

Another argument which opposes the development of artificial life forms supports that such type of experiments may create a reductionist conception of life and its value [23]. This critique is based on a fear that scientists tend to follow a mechanistic approach of nature and make descriptions of biological phenomena to look like a series of chemical interactions obeying mathematical equations. Yet, the majority of scientists reject the idea that life is just a sum of chemical substances interacting with each other—they do not conceive the whole of a living organism as a sum of its parts. In our opinion, it is quite improbable that this view will change in the future, as the more we discover about life the more we realize that there is more to it than that and we remain ignorant of many of life’s mysteries [28].

In sum, we understand that realizing humanity’s present capacity to take such a big step towards comprehending and controlling some of the mechanisms of life might inspire sentiments of fear, especially given the history of uses of scientific discoveries and innovations (gunpowder, nuclear weapons), yet it is in our hands to control the way scientific knowledge might be used. In other words, we find that as with every other technological advancement, it is the use that might be immoral, not the technology itself.

### 3.4. Virtue Ethics and Life Synthesis

Apart from the fear of hubris or disrespect towards God or nature which, for some, makes life synthesis intrinsically bad (a deontological perspective), or a belief that such experiments will lead to dangerous paths, creating new weapons or threatening the environment (a more consequentialist approach), a type of virtue theory ethics also disapproves this kind of research [15]. This view emphasizes the importance of the virtues which must be cultivated, namely humility, gratefulness for the giftedness of life, respect for the laws of nature, and precaution in front of the unknown consequences, which may lead to an abstention from the use of all the technological means humanity has under its disposition. According to the teleological point of view of virtue ethics, the manipulation of life alienates our species from the universal telos of shared existence—it generates a conception of “sheer thinghood” for living beings and separates humanity from the other species, creating an “us and them” [15]. This attitude towards nature neglects the fact that we are part of an ecosystem and tries to bring every aspect of the environment under control for the maximization of utility.

Summarizing, one can find that the arguments opposing the current and future projects of synthetic biology draw from the vocabulary and theoretical basis of all three basic moral theories. We must also acknowledge the existence of an intuition among the wider public against the synthesis of life forms. It has been pointed out that artificial life brings humanity to a new place in its relationship with nature, and that the “living machines” are a new adjustment in the ecosystem in the sum of morally significant entities [29,30,31]. In the next part we will try to provide an answer to the arguments presented and develop our own approach, promoting the permissibility of the creation of synthetic life under specific terms.

### 3.5. Difference between Artificial “Copies” and Natural Beings

One of the main sources of concern towards creating life is the fact that the new organism’s synthesis out of non-living parts (its artificiality) will constitute a breach with the natural world. We mentioned that part of our connection to the ecosystem is our common ancestry—humans constitute a part of the sum of living beings of planet Earth, they are beings which emerged after millions of years of evolution. For some, when creating living organisms, humanity bypasses natural selection and makes scientific will superior to natural evolution, acting thus in a hubristic way. In order to respond to this argument, we need to distinguish between a potential creation of artificial copies of existing organisms and a synthesis of completely new types of organisms. As far as copies are concerned, one must underline that the existence of an identical—artificially-generated—organism carries no special moral weight. In order to discriminate between the natural organism and its copy one must prove that the different way they came into existence (synthesis or birth) is morally significant. We find that a copy does not constitute a breach in the chain of evolution as it is identical with the natural entity. By copying nature, humanity does not prove its superiority towards it but rather expresses admiration and curiosity for its complexity. We base this argument on the assumption that if one can spot no difference between a “copied” artificial life form and the natural “original”, one cannot discriminate between the two [32]. If one would create, for example, a jellyfish identical to a natural one, there is no sufficient moral reason for us to judge that one—the natural—is better than the other. If this were the case, one would tell us that the so-called “copy” is, in reality, a natural jellyfish, and vice versa. In particular, we would have to change the way we value the animal accordingly, something which would be absurd.

There can also be the case of a potential creation of an organism which will externally resemble a natural one but will have different properties, for example, a jellyfish created to be used as a biosensor to detect high levels of pollution by changing color when exposed to a specific substance. In that case, these organisms constitute an almost entirely different type of entity—it will be an organism which must matter morally according to its complexity and not be treated as a mere instrument.

We argue that the potential creation of an organism which resembles a natural one must be regulated taking into account the level of its biological complexity, as one may argue that it does not carry the same moral worth as its “natural” twin.

On the other hand, scientists may be considering the possibility of creating entirely new life forms, as has happened in the past with hybrids, which were generated by humans through breeding. At the time however, human capabilities in animal-crossing were limited due to the knowledge of genetics. In this new era, genetic technology has given scientists the power to create chimeras and make the first steps towards the synthesis of life. Thus, it is crucial to regulate the terms under which potential new life forms may come into existence. For these reasons, we need to consider whether the creation of entirely new organisms is morally significant, if the creation of new species causes negative intuitions, and if so, for what reasons [33].

## 4. What Kind of Organisms Shall We Create?

In examining this topic, we claim that the thin line between artificial–natural must be conserved in order to better understand the way in which a being comes to life and make the distinction between an organism created from scratch and an organism generated through natural reproduction. However, at the same time, as mentioned, we find that this distinction is morally insignificant as far as the treatment of these organisms is concerned. We have argued that the moral status of an organism remains the same if it is a copy of an existing species, but on what terms does a completely new life form (an XNA organism for example) obtain its status?

We find that organisms complex enough to be considered morally—in other words multicellular conscious beings—would rather not be created. In our opinion, the more complex an organism is, the more difficult it is to ignore its immanent aims and its interests for self-sustainability and pain avoidance. A potential cause of suffering to a sentient being by its scientist/creator may significantly harm these types of organisms’ interests and, as we consider them to matter morally, we prefer not to put the creators in the position in which they may harm the new being. For this reason, its creation must be strictly regulated [34] for aims generally judged as superior, such as research for health issues of more complex organisms and environmental sustainability. We are critical of the creation of entirely new complex organisms, not only for reasons of biosafety and security but also for moral reasons. We understand the complexity of the term consciousness, thus we choose a more biological approach in our effort to specify it. We also stress the need for up to date legislation with research concerning levels of consciousness in living beings [35,36]. Simpler life forms, such as viruses, bacteria or protozoa, could be generated according to safety and security regulations.

### Difference between Artificial “Copies” and Artificial New Species

What makes an artificial multicellular animal-like organism different to a lab mouse born in order to be used for experimental reasons is that, although this specific mouse was created or born in order to be used in an experiment (and therefore is quasi-objectified), being part of an experiment is not an inherent characteristic of its nature. Mice are not naturally lab animals, designed for research purposes. It could very well be released without that making any difference to the aim of its existence: to survive and reproduce. The same goes for an artificial mouse. On the other hand, a new artificial entity will always be partially instrumental, created as a lab organism—an object for experimental use. Even if one believes that the ecosystem, or organisms in particular, have no specific “telos” and therefore a natural or artificial organism has no specific purpose, one should consider that in that case that artificial new life forms differ from the natural and their artificial copies in that they do have a purpose—they were created for a reason.

One can think that an artificial mouse generated to be part of an experiment and an artificial animal-entity generated for the same reason are similar, as they are both synthetic and both are used in a lab. In that case, what is the difference between a synthetic mouse which, as we argued, carries the same moral value as a natural one, and an artificial animal entity designed and created for experimental reasons? Is it mere appearance, an issue of DNA?

We already have legislation preventing harm to animals in research and regulating their use in experiments [37]. An artificial being may have moral significance based on the fact that it resembles a natural being. An entirely new life form on the other hand may draw its moral status from its complexity, its level of consciousness. A living being will always develop its own goals: to self-sustain and reproduce. Moreover, a complex multicellular organism capable to feel pain and agony and conscious of a part of its identity or at least able to create a primary concept of a “self” [38], will also develop further interests, such as avoiding circumstances which may cause pain. One needs to find a strong moral reason why it should be manipulated in a way which may be contrary to its will. Such a reason cannot be but experiments concerning subjects of higher moral value, such as the ones addressing health or environmental problems.

We believe that the use of (natural) animals for experimental reasons is morally problematic and should be avoided if possible. For the same reasons, the creation of beings complex enough to feel pain and agony in order to experiment with constitutes a moral step backwards. What we should aim to do is avoid causing pain and suffering to anything and not just change the object of our potentially painful operations.

A crucial issue which might emerge in the synthesis of a complex moral being is that its creators may argue that it might be used in order to save a human life, through organ transplantation for example. In that case, we believe that a generation of such an entity is also morally problematic: to generate life in order to use or even destroy it is by itself morally impermissible. It will be different than a case of xenotransplantation (which by itself is a controversial subject). We find that this use may lead to reductionism, reducing living beings into sums of biologically functional parts.

## 5. Conclusions

We have argued that synthetic biology is not a novel scientific discipline, it emerged from the development of biotechnological research under the influence of the systemic biology’s approach and the scientific and engineering tools which were developed during the Human Genome Project research. In addition, amongst the various research strategies used in synthetic biology, only the bottom-up approach can be related with the construction of artificial synthetic life forms. Secondly, we presented an opinion in favor of the evolution of technologies permitting the creation of synthetic life forms and claimed that synthetic beings possess moral status. On the other hand, we disapproved the potential creation of multicellular complex and conscious beings for reasons other than scientific research concerning human health or environmental sustainability, as we support the idea that this type of organisms’ status does not permit ignoring their interests and/or causing unnecessary pain. We believe that the objectives of synthetic biology in general, and life –synthesis in particular, must be the promotion of humanity’s health and the protection of the environment, and hope for a just and sustainable distribution of scientific benefits. Although the science of biology has entered a new era, we must not abandon principles such as the respect of life and dignity, which lead us so far. Biologists need to remember that justice and virtue is what separates “science from roguery” [39].
